# Anterior Cruciate Ligament Reconstruction: Is Biological Augmentation Beneficial?

**DOI:** 10.3390/ijms222212566

**Published:** 2021-11-22

**Authors:** Emerito Carlos Rodríguez-Merchán

**Affiliations:** 1 Department of Orthopedic Surgery, La Paz University Hospital—IdiPaz, 28046 Madrid, Spain; ecrmerchan@hotmail.com; 2 Osteoarticular Surgery Research, Hospital La Paz Institute for Health Research—IdiPAZ (La Paz University Hospital—Autonomous University of Madrid), 28046 Madrid, Spain

**Keywords:** anterior cruciate ligament, reconstruction, biological augmentation, results

## Abstract

Surgical reconstruction in anterior cruciate ligament (ACL) ruptures has proven to be a highly effective technique that usually provides satisfactory results. However, despite the majority of patients recovering their function after this procedure, ACL reconstruction (ACLR) is still imperfect. To improve these results, various biological augmentation (BA) techniques have been employed mostly in animal models. They include: (1) growth factors (bone morphogenetic protein, epidermal growth factor, granulocyte colony-stimulating factor, basic fibroblast growth factor, transforming growth factor-β, hepatocyte growth factor, vascular endothelial growth factor, and platelet concentrates such as platelet-rich plasma, fibrin clot, and autologous conditioned serum), (2) mesenchymal stem cells, (3) autologous tissue, (4) various pharmaceuticals (matrix metalloproteinase-inhibitor alpha-2-macroglobulin bisphosphonates), (5) biophysical/environmental methods (hyperbaric oxygen, low-intensity pulsed ultrasound, extracorporeal shockwave therapy), (6) biomaterials (fixation methods, biological coatings, biosynthetic bone substitutes, osteoconductive materials), and (7) gene therapy. All of them have shown good results in experimental studies; however, the clinical studies on BA published so far are highly heterogeneous and have a low degree of evidence. The most widely used technique to date is platelet-rich plasma. My position is that orthopedic surgeons must be very cautious when considering using PRP or other BA methods in ACLR.

## 1. Introduction

**Figure 1:**
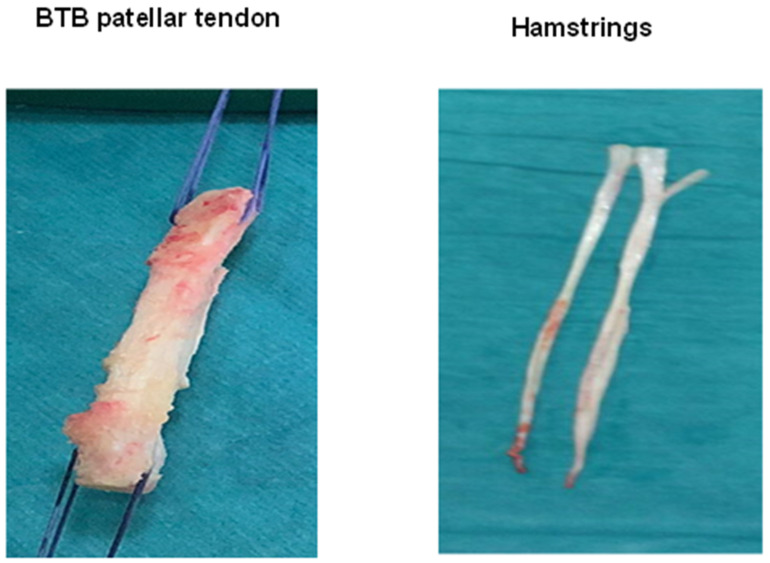
Types of tendon autografts for anterior cruciate ligament reconstruction (ACLR): bone-tendon-bone (BTB) patellar tendon autograft (**left**); hamstrings autograft (**right**).

Anterior cruciate ligament (ACL) ruptures are frequent injuries. In the United States, an estimated 200,000 such ruptures occur annually [1]. ACL reconstruction (ACLR) is the undisputed surgical technique of choice, especially for young active patients (Figure 1) [2]. The ACLR technique has been improving in recent years [3] but is still imperfect. Patients who undergo ACLR almost never recover their initial knee function [4], and there is also the risk of ACLR rupture and early osteoarthritis in the operated knee [5,6].

The outcome of an ACLR depends on the graft healing response, i.e., osseointegration at the graft-tunnel interface and remodeling of the intra-articular graft, known as “ligamentization” [7]. The native ACL is attached to the bone by a direct fibrocartilaginous enthesis, whose morphological structure consists of four zones, with a gradual transition from tendon to cartilage and from mineralized cartilage to bone [8]. After ACLR, however, the graft heals through an indirect enthesis formed by fibrovascular scar tissue, which is biomechanically of poorer quality than the previously mentioned direct enthesis [9]. Therefore, techniques are being investigated that can biologically enhance the healing of the graft used in ACLR and thereby facilitate early and intensive postoperative rehabilitation and a faster return to the physical activity level prior to the ACL rupture [10].

In 2021, Sherman et al. presented the results of a survey on global trends in ACLR conducted with the members of the Anterior Cruciate Ligament Study Group, showing that 21% of them employed some type of biological augmentation (BA) in primary ACLR [11].

The aim of this article is to answer the following question: is BA necessary in ACLR? To this end, we conducted a narrative review of the most recent literature on the subject.

## 2. Types of Biological Augmentation Techniques in Anterior Cruciate Ligament Reconstruction

**Figure 2:**
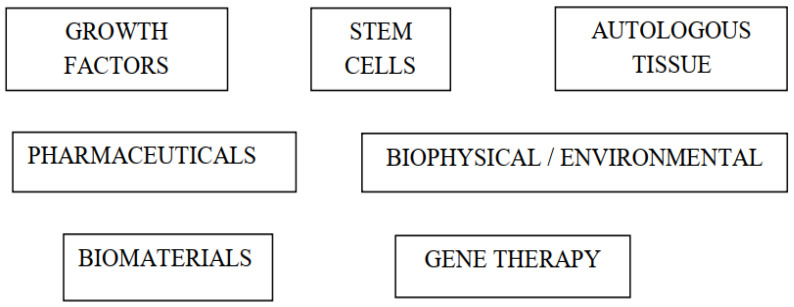
Biological augmentation methods used in anterior cruciate ligament reconstruction (ACLR).

There are several types of BA techniques that have been used in ACLR (Figure 2). Platelet-rich plasma (PRP) is the most studied technique to date, although the published clinical results are conflicting, and the methodological quality of these publications has been suboptimal. Other biological techniques studied in clinical trials include remnant-augmented ACLR, bone substitutes, calcium phosphate-hybridized grafts, extracorporeal shockwave therapy (ESWT), and adult autologous non-cultivated stem cells [12].

### 2.1. Animal Studies

The most investigated BA techniques to date in animal studies are biomaterials, growth factors, stem cells, autologous tissue, gene therapy (genetically modified stem cells), pharmaceuticals, biophysical interventions, and combinations of these techniques. According to Hexter et al., the methodological quality scores of the animal studies ranged from 4/8 to 8/8, with 97% of the publications scoring at least 5 points (considered the threshold for acceptable methodological quality) [12].

### 2.2. Clinical Studies

In the study by Hexter et al., 80% of the publications evaluated growth factors, all of them in the form of platelet concentrates, such as PRP. The rest studied platelet-rich fibrin matrices, autologous serum, platelet leukocyte gels, and autologous platelet concentrates. In most publications, the BA technique was employed during the surgical procedure, although a number of studies used the technique postoperatively. Postoperative treatment with shock waves, joint injections of growth factors in the form of PRP, and autologous serum have also been investigated [12].

According to Hexter, most studies related to BA of ACLR have focused on grafting, tunneling, and the intraoperative application of biological material. However, a number of studies have focused specifically on bone tunnels. Approximately 50% of the publications reported positive results; these included studies on platelet concentrates, calcium phosphate hybridized tendons, and ESWT. A number of clinical studies using remnant repair and bone substitutes have shown mixed results, with positive radiological results but no difference in clinical outcomes. Studies on platelet concentrates and bone marrow-derived mesenchymal stem cells (BMSCs) have shown no beneficial effects for the BA technique [12]. In terms of the quality of the clinical studies, 75% of the studies were randomized controlled trials, and 25% were non-randomized studies (case-control studies and prospective cohort studies). The methodological quality of the clinical studies is generally considered poor [12].

## 3. Growth Factors

Most publications refer to growth factors, used individually or as platelet concentrates [13] (Table 1).

The growth factors investigated in animals include bone morphogenetic protein (BMP), epidermal growth factor, granulocyte colony-stimulating factor, basic fibroblast growth factor, transforming growth factor-β (TGF-β), hepatocyte growth factor, and vascular endothelial growth factor [12]. Platelet concentrates, which are sources of bioactive molecules and multiple growth factors, such as platelet-derived growth factor, TGF-β, and vascular endothelial growth factor, include PRP [13], fibrin clot [14], and autologous conditioned serum [15].

In 2020, Momaya et al. evaluated the cost of PRP and stem cell injections and determined that the mean cost of a PRP injection in the United States was USD 707 (range, USD 175–USD 4973), and the mean cost of a stem cell injection was USD 2728 (range, USD 300–USD 12,000) [16].

Growth factors are thought to enhance the healing process and can be used to target graft healing, both on the graft-tunnel interface and to promote intra-articular ligamentization [17].

## 4. Stem Cells

Numerous types of mesenchymal stem cells (MSCs) have been reported to enhance healing at the graft-tunnel interface in experimental studies (animal models). Such cells can be isolated during surgery through bone marrow aspiration or cultured prior to surgery [18]. Table 2 summarizes the different types of stem cells used in ACLR [18,19,20,21,22,23,24,25,26].

## 5. Autologous Tissue

Current evidence indicates that when the ACL ruptures, locally released CD34+ vascular MSCs contribute to the rupture’s healing [18]. An important advantage of employing autologous tissue rather than cultured MSCs is the ability of autologous tissue to provide a source of regenerative cells without risk of rejection or malignancy. The goal of attaching the ACL remnant to the graft is to promote its ligation. Most animal studies on this subject have shown positive results, although some found no differences [27,28].

In a series of 128 patients, Ouanezar et al. observed that ACLR using the single anteromedial bundle biological augmentation technique significantly improved clinical and functional outcomes. The mean follow-up was 31.7 months (range, 24–44.3). Twenty-four (18.7%) revision surgeries were required: 10 meniscal procedures, 7 ACL revisions, 5 arthroscopies for cyclops lesions, 1 microfracture, and 1 manipulation under anesthesia. The comparative laxity between the healthy and operated sides and the rates of reoperation, graft failure, and cyclops lesions were not significantly different between the ≥50% or <50% ACL remnant groups [29].

A published study indicated that the periosteum is a source of regenerative cells that can promote osteogenesis and chondrogenesis; that is, the use of periosteal cell graft healing could theoretically recreate a direct type of fibrocartilaginous enthesis [30]. Table 3 summarizes the types of autologous tissues used in ACLR.

## 6. Pharmaceuticals

A number of drugs that could modulate the inflammatory response after ACL rupture and enhance graft healing have been investigated in several animal models (Table 4).

Using rabbits undergoing bilateral ACLR injected with a matrix metalloproteinase-inhibitor alpha-2-macroglobulin in the knees, Demirag et al. studied the role of this inhibitor in graft healing. At 5 weeks’ follow-up, the knees in which the inhibitor was injected showed a more mature graft-tunnel interface, a decrease in matrix metalloproteinases in the synovial fluid, and improved biomechanical strength [31].

The effect of bisphosphonates on osseointegration after ACL rupture has also been studied in a rat model of ACLR. At 6 weeks of follow-up, the local or systemic administration of alendronate resulted in improved bone tunnel mineralization, decreased bone loss in the peri-tunnel, and increased graft integration in the tunnel [32].

In a study comparing rats treated with daily subcutaneous parathyroid hormone against control rats, computed tomography scans showed greater thickness and better trabecular bone microarchitecture in the parathyroid hormone-treated animals than in the control animals treated with saline injections [33].

## 7. Biophysical and Environmental

In a study of rabbits, Yeh et al. used hyperbaric oxygen to improve graft neovascularization. Animals treated with hyperbaric oxygen showed improved neovascularization, osseointegration, and biomechanical properties [34].

A number of published studies on animal models have reported positive results with the use of low-intensity pulsed ultrasound [35]. One animal model (rabbits) study observed that animals treated with low-intensity pulsed ultrasound had better ligamentization, a finding attributed to the upregulation of genes such as TGF-1 [35].

In a rabbit model, Wang et al. observed that ESWT applied to the tibial tunnel immediately after ACLR improved healing at the graft-tunnel interface, both histologically and biomechanically, at 8 and 24 weeks of follow-up [36]. Table 5 summarizes the biophysical and environmental methods used in ACLR.

## 8. Biomaterials

This section will discuss the four key technologies related to biomaterials: biological fixation methods, biological coatings, biosynthetic bone substitutes, and osteoconductive materials (Table 6).

### 8.1. Biological Fixation Methods

A number of experimental studies on ACLR performed in rabbits have shown that magnesium-based interference screws can accelerate graft mineralization, promote bone formation in the periscrew region, and improve extensor tendon osseointegration [37,38]. In a rabbit model, the use of biodegradable polylactide bolts as the bone anchor and poly(D,L-lactide-co-glycolide) nanofibrous membranes at the graft-tunnel interface has been shown to improve bone growth and reduce peri-tunnel bone loss [39].

### 8.2. Biological Coatings

Studies have shown that the graft-tunnel interface healing of artificial grafts can be improved by using various types of coatings, including chitin [40], bioglass [41], gelatin, and hyaluronic acid [42], polystyrene sodium sulfonate [43], and collagen matrix [44]. Similar coatings have been used with tendon autografts. In a rabbit model, for example, hydroxyapatite-doped polycaprolactone nanofiber membrane wrapped around autograft hamstring tendons improved their tissue integration and mechanical strength [45].

### 8.3. Biosynthetic Bone Substitutes

Animal studies have shown positive results using biosynthetic bone substitutes, such as demineralized bone matrix (DBM) and recombinant bone xenograft. These biomaterials appear to improve graft tunnel interface healing due to their osteoinductive and osteoconductive properties [46]. DBM has been shown to improve tendon-bone healing in animal models of rotator cuff pathology. DBM is manufactured by the acid extraction of the mineral component of bone, which leaves a collagen scaffold containing growth factors such as BMPs [47,48,49]. Applying DBM to the tendon-bone interface in ACLR appears to increase tendon-bone healing in rabbit [46] and rat models [50].

### 8.4. Osteoconductive Materials

Calcium phosphate (CaP) is a resorbable and osteoconductive biomaterial that has shown positive results in several animal models for assessing graft healing [51]. Furthermore, this positive effect has been reported to be enhanced by incorporating strontium [52]. In an experimental study on goats, Mutsuzaki et al. investigated a CaP-hybridized flexor tendon. The authors observed that at 6 months, the animals treated with the CaP-hybridized flexor tendon had better knee stability and greater osseointegration than the untreated goats at the graft-tunnel interface [51].

## 9. Gene Therapy

Most of the recently published studies on gene therapy to improve graft healing in ACLR have been based on MSCs [53,54]. In this method, stem cells are transfected with growth factors such as BMP2, platelet-derived growth factor subunit B and TGF-β. By delivering a continuous and stable concentration of these growth factors to the graft healing site, graft healing in ACLR can be improved [55]. In contrast to the local intraoperative application of growth factors, genetic modification of stem cells offers the advantages of a strong and sustained effect of growth factors. In a rabbit model of ACLR, Chen et al. observed that implantation of genetically modified MSCs with basic fibroblast growth factor and BMP2 at the graft-tunnel interface led to improved cellularity, better new bone formation, and improved mechanical properties. Compared with single gene therapy, the co-application of these 2 genes was more powerful and efficient [53]. Table 7 summarizes the types of gene therapy used in ACLR.

## 10. Molecular Insights in ACLR

**Figure 3:**
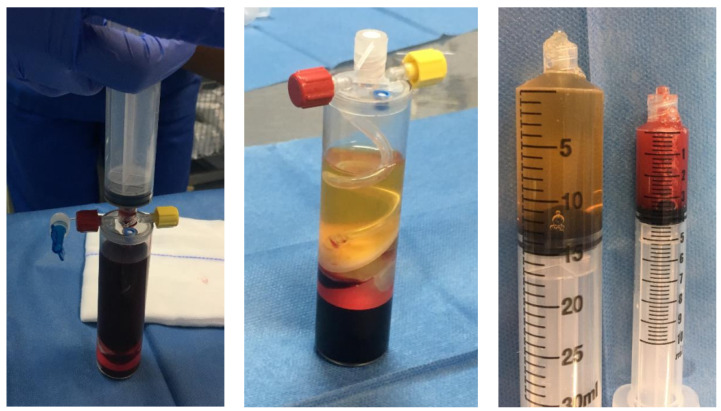
Platelet-rich plasma (PRP) kit-30 mL blood extraction. Platelet poor plasma (**left**), platelets and white blood cells (Buffy Coat) (**center**), red blood cells (**right**).

According to Hexter et al., goals for augmentation of ACLR comprise easing graft to-bone healing, optimizing the ligamentization process, and rendering further stability while the graft is temporarily frail during remodeling. Several growth factors and bioactive molecules are encountered in a number of platelet preparations such as PRP, fibrin clot, and autologous conditioned serum [12]. PRP is the most commonly used so far in ACLR (Figure 3).

Some of these, comprising PDGF, VEGF, and TGFβ, have been involved in both graft-to-bone healing and graft maturation and remodeling. Platelet preparations have been the topic of many clinical studies trying to augment these processes, but the outcomes remain variable and unverified. In a few prospective randomized controlled studies, intraoperative local administration of PRP gel to the graft and tunnels was associated with better healing characteristics on postoperative MRIs when compared with controls [56,57,58].

Radice et al. found that ACLR augmented with PRP accomplished intra-articular segment signal homogeneity on T1- and T2-weighted MRI sequences in 48% of the time needed by the control group (*p* < 0.001), suggesting that PRP might have hastened the graft maturation process [56]. Rupreht et al. noticed data compatible with augmented vascular density and microvessel permeability in the proximal tibial tunnel at 1 (*p* = 0.019) and 2.5 months (*p* = 0.008) postoperatively, suggesting a positive influence on graft-to-bone healing and incorporation [57].

Vogrin et al. encountered a significantly higher level of vascularization on contrast-enhanced MRI in the osteoligamentous interface of the PRP group when compared with the control group at 4–6 weeks [58]. Seijas et al. attained analogous outcomes in a randomized trial with nonselective intra-articular administration of PRP injected percutaneously into the suprapatellar space after portal closure, with significantly higher stages of remodeling observed on postoperative MRIs at 4, 6, and 12 months [59].

However, Orrego et al. noticed an isolated enhancing impact on the graft maturation process without a difference at the graft–bone interface with application of a platelet concentrate intraoperatively [60]. Vadala et al. observed that direct administration of PRP into both femoral and tibial tunnels was not efficacious in speeding up graft to bone integration or averting tunnel enlargement [61]. Mirzatolooei et al. found no difference neither in tunnel widening between PRP injection groups and controls on postoperative advanced imaging, nor in laxity on clinical examination at 12 weeks [62].

In a randomized controlled trial, 1 year after ACLR the administration of PRP was related to a decrease in swelling 24 h after the surgical procedure. However, no difference in the IKDC scores or radiologic graft healing between PRP and control groups was found [63]. Komzak et al. encountered no difference in the functional scores between patients and controls in a prospective study evaluating the impact of PRP on graft healing [64].

Given the relative non-specificity and mixed clinical outcomes of platelet-based therapies, some authors have utilized alternative more targeted procedures.

The exposed nature of the intra-articular portion of the ACLR graft has made that some authors think that the addition of a scaffold may ameliorate the effectiveness of bioaugmentation with growth factors and platelet preparations. For example, porous collagen scaffold carriers may diminish plasmin-mediated degradation of fibrin in PRP [65].

Berdis et al. analyzed 109 knees in 101 adolescent patients in whom hamstring ACLR was carried out with bioaugmentation with PRP contained in a porous bovine collagen matrix carrier (TenoMend; Exactech, Ramsey, NJ, USA). Most patients (132, 92%) returned to their preinjury level of competition, while 7 (55) patients sustained a reinjury requiring revision surgery. These authors thought that these outcomes compared favorably with the 25% rate of reinjury and revision among pediatric and adolescent athletes published by other authors [66,67]. One patient evaluated with second-look arthroscopy for a new injury at 7 months after the initial reconstruction demonstrated complete ligamentization and neovascularization of the graft [66]. It is appropriate to mention here the systematic review and meta-analysis reported by Wiggins et al [68]. It showed that 1 in 4 young athletic patients who sustain an ACL injury and return to high-risk sport will go on to sustain another ACL injury at some point in their career, and they will likely sustain it early in the return-to-play period. In order to help young athletes reintegrate into sport more safely and reduce second injury, activity modification, improved rehabilitation, return-to-play guidelines, and the use of integrative neuromuscular training are paramount [68].

Augmentation with amnion-based matrices might render an option to collagen scaffolds that already have helpful growth factors and bioactive substances [69]. Woodall et al. have described a technique for augmentation of soft-tissue ACLR utilizing Amnion Matrix Thick graft (Arthrex, Naples, FL, USA) [70]. Lavender and Bishop have added a bone marrow composite graft to the tunnels and injecting the amnion-wrapped graft with bone marrow concentrate, and eventually augmenting the assemble with a suture tape brace [70]. A clinical trial to evaluate ACLR augmented with an amnion wrap and bone marrow aspirate was registered in September 2017, but otherwise, no results have been published for these reconstruction bioaugmentation procedures [71].

A recently published study on a sheep model of ACLR has once again demonstrated that BMSCs and PRP significantly improve graft maturation [72]. Although 75% of clinical studies on BA have evaluated the efficacy of PRP, evidence demonstrating this efficacy is lacking. In addition, most clinical studies on BA have not met the minimum reporting standards. Biomaterials have been investigated the most in ACLR but mainly in animal models (90%), whereas only 10% have been clinical studies. Future clinical studies should analyze the role of biomaterials that are already employed in clinical practice, such as DBM, which has been shown effective in animal models [73]. Further analysis of the role of periosteum, which has demonstrated efficacy in animal models, is needed [74].

In 2020 Yu et al. have introduced and validated a metal-free, reproducible, and reliable mouse model of ACLR surgery as an efficacious method for a better understanding of molecular mechanisms of graft-tunnel healing after ACLR [75]. One hundred and fifty C57BL/6 mice were randomly allocated into five groups: Group 1 (mice with intact ACL), Group 2–4 (mice underwent modified ACLR surgery and sacrificed 1-, 2-, and 4-weeks after surgery), and Group 5 (mice underwent unmodified ACLR surgery and sacrificed 4 weeks after surgery). Micro-computed tomography (CT), biomechanical histological, and immunohistochemical (IHC) analyses were carried out to characterize the modified ACLR. Micro-CT analysis showed there was a non-significant increase in BV/TV and BMD of the bone tunnel during the tendon-to-bone healing following ACLR. Biomechanical tests demonstrated that the mean load-to-failure forces of Group 3 and 4 are equal to 31.7% and 46.0% of that in Group 1, while the stiffness was 33.1% and 57.2% of that of Group 1, respectively. No obvious difference in biomechanical parameters was found between Groups 4 and 5. Histological analysis showed that formation of fibrovascular tissue in the tibial tunnel and aperture in Groups 4 and 5 and direct junction appeared between tendon graft and tunnel both in Groups 4 and 5. IHC results demonstrated that there are gradually enhanced expressions of Patched1, Smoothened and Gli2 concomitant with diminished Gli3 protein in the tendon-bone interface during the tendon-bone healing process. This mouse model could be utilized to investigate the detailed molecular mechanisms of graft-tunnel healing following ACLR [75].

According to Atherton et al. the utilization of the vancomycin wrap to pretreat the hamstring graft in ACLR has significantly diminished rates of postoperative infection. However, it was not known whether this antibiotic treatment (5 mg/mL concentration) affects the molecular composition of the graft. In a controlled laboratory study, surplus hamstring tendon collected after routine ACLR surgery was utilized for in vitro cell culture and ex vivo tissue experiments [76]. Vancomycin was utilized at 5 mg/mL in RPMI or saline diluent to treat cells and tendon tissue, respectively, with diluent control conditions. Cell viability at 30, 60, and 120 min was evaluated via colorimetric viability assay. Tendon cells treated with control and experimental conditions for 1 h was assessed utilizing semiquantitative reverse transcription analysis, immunohistochemistry staining, and protein quantitation via enzyme-linked immunosorbent assay for changes in apoptotic, matrix, and inflammatory gene and protein expression. Vancomycin treatment at 5 mg/mL significantly reduced tenocyte viability in vitro after 60 min of treatment (*p* < 0.05); however, this was not sustained at 120 min. Vancomycin-treated tendon tissue demonstrated no significant increase in apoptotic gene expression, or apoptotic protein levels in tissue or supernatant, ex vivo. Vancomycin was associated with a decrease in inflammatory proteins from treated tendon supernatants (IL-6; *p* < 0.05). Vancomycin ACL wrap did not alter the molecular structure of the ACL hamstring graft and may ameliorate graft integrity [76].

## 11. Discussion

All BA methods analyzed in this article have shown good outcomes in animal studies; however, the clinical studies on BA published so far are highly heterogeneous and have a low degree of evidence.

Iorio et al. reported a randomized controlled trial with 40 patients examining the clinical and radiographic impact of hamstring autograft augmentation with nanohydroxyapatite to ease graft-to-bone healing. Lysholm, Tegner, IKDC scores, and KT-1000 arthrometer readings, did not differ significantly between the study group and the control group. However, radiographic parameters related to graft strength, interface incorporation, and bony remodeling showed a tendency toward better outcomes with nanohydroxyapatite augmentation [77].

In two different randomized controlled trials with minimum 2-year follow-up, Mutsuzaki et al. found better outcomes after ACLR with calcium phosphate hybridized hamstring autograft. Significantly better Lysholm scores at 2-year follow-up were found with calcium phosphate- hybridized hamstring autograft compared with controls, as well as significantly less laxity on KT-1000 arthrometer testing at 1 and 2 years postoperatively, and significantly less bone tunnel enlargement in both the femur and tibia. Therefore, calcium phosphate hybridization was shown to avert bone tunnel enlargement in anatomic hamstring autograft ACLR. However, the clinical significance of this finding remains uncertain [78,79].

In 2021, Bailey et al. analyzed the effect of intraoperative PRP on postoperative knee function and complications after ACLR with meniscal repair at 2 years of follow-up. In a level 3 evidence study (ClinicalTrials.gov identifier: NCT03704376, accessed on 7 November 2021), Bailey et al. observed that adding intraoperative PRP did not improve the self-reported knee function, functional performance, or time to return to the preinjury activity level for patients undergoing ACLR with meniscal repair. The use of PRP also had negative consequences in terms of the postoperative recovery of knee range of motion. Based on these data, the authors stated that orthopedic surgeons should cautiously consider the application of PRP during surgery for intra-articular knee injuries [18]. In a systematic review and meta-analysis published in 2021, Lv et al. concluded that the use of PRP could significantly decrease pain in the short term but not in the long term [80].

The recently published survey by Sherman et al. concluded that the vast majority of ACL-SG members (90%) used a single-bundle technique for ACLR and that more than 50% chose hamstring autograft as the primary graft of choice [11]. More than 80% of ACL-SG members considered that primary ACLRs should be performed in conjunction with extra-articular augmentation. Fifty percent of ACL-SG members preferred bone-tendon-bone autograft for revision ACLR, while extra-articular augmentation was employed more frequently in revision ACLR than in primary ACLR. More than 50% of ACL-SG members routinely used a brace after ACLR. The minimum time frames for returning to pre-injury sports after primary ACLR were 6–8 months for 44% and 8–12 months for 41% [11].

It is important to highlight the discrepancy between 2 recent articles: one on an experimental study stating that orthobiologics can accelerate biological events related to tendon allograft incorporation [72] and another on a clinical study on meniscal repair in the ACLR setting stating that the added use of intraoperative PRP does not improve the analyzed parameters (self-reported knee function, functional performance, and time to return to previous activity). According to Hexter et al., the use of PRP might have negative consequences for the postoperative recovery of knee range of motion [18].

In the sheep model described by Hexter et al. in 2020, the authors observed that BMSCs and PRP significantly enhanced graft maturation, indicating that orthobiologics might accelerate biological events related to tendon allograft incorporation [72]. They also found that femoral tunnel expansion was significantly correlated with inferior maturation of the intra-articular graft. The clinical relevance of this study was that BMSCs and PRP enhance allograft healing and that biological modulation of graft healing can be assessed by magnetic resonance imaging. Fifteen sheep underwent ACLR with unilateral tendon allograft using aperture fixation and were randomized into 3 groups (*n* = 5). Group 1 received 1 × 10^7^ allogeneic BMSCs in 2 mL of fibrin seal; group 2 received 12 mL of PRP in a plasma clot injected into the graft and bone tunnels, and group 3 (control) received no adjunctive treatment. At autopsy performed at 3 months, a graft maturation score was calculated by summing the graft integrity, synovial coverage and vascularity, graft thickness and apparent tension, and synovial sealing at the tunnel apertures. The signal-noise quotient (SNQ) and fibrous interzone were analyzed by magnetic resonance imaging (*n* = 2 animals per group) to assess intra-articular graft maturation and tendon-bone healing, respectively. Spearman’s rank correlation coefficient (r) of the SNQ, the graft maturation score at autopsy, and the bone tunnel diameter were analyzed. The BMSC group (*p* = 0.01) and the PRP group (*p* = 0.03) obtained a significantly higher graft maturation score than the control group. The BMSC group scored significantly higher for synovial sealing at the tunnel apertures (*p* = 0.03) than the control group. The graft maturation score at autopsy correlated significantly with the SNQ (r = −0.83, *p* < 0.01). Femoral tunnel diameter at the opening (r = 0.883, *p* = 0.03) and mid-portion (r = 0.941, *p* = 0.02) were positively correlated with the SNQ [70].

In contrast, the retrospective matched case-control study published Bailey et al. analyzed 324 patients (162 PRP patients and 162 control patients) and concluded that the added use of intraoperative PRP did not improve the analyzed parameters [18]. Patients were matched for age, sex, graft type, and meniscal lesion. A single assessment numeric evaluation (SANE) was administered at 2 years, and lesion surveillance was performed. Secondary outcomes included return-to-activity time (months), self-reported knee function (International Knee Documentation Committee score), functional performance tests (knee range of motion, single-leg balance, single-leg hopping, agility tests), and postoperative complications (graft failure, infection, loss of mobility requiring repeat arthroscopy to remove adhesions, venous thrombosis, etc.). At the 2-year follow-up, no differences in SANE knee function scores were found between the PRP and control groups. In addition, no differences were observed between the groups in self-reported function, functional performance tests, and time to return to activity. The PRP group showed a higher frequency of postoperative range-of-motion loss than the control group (13.6% vs. 4.6%; *p* < 0.001). No other differences in postoperative complications were observed [18].

Regarding the use of BA in orthopedic sports surgery, Looney et al. stated that although the diversity and availability of new biologic technologies continue to increase, the literature remains inconclusive as to the optimal indications for its use [81]. Long-term results have led to the increasing recognition of the limitations related to the surgical treatment of ACL ruptures; BA could therefore help in this regard. However, BA should not be considered a panacea. Orthopedic surgeons should maintain realistic expectations regarding the capabilities of BA and not lose sight of other factors that might contribute to poor outcomes in certain patients. For example, BA will never solve limb alignment problems, which should be previously addressed by osteotomy [81].

In a retrospective review, McMillan et al. analyzed 14 patients operated on for ACLR using an augmented quadruple-stranded hamstring allograft with a type I resorbable collagen matrix impregnated with PRP. The authors observed that their technique produced good early clinical results (at the 2-year follow-up) and had no ACLR failures. This novel ACLR approach could improve the incorporation of biologic grafts into host bone tunnels [82]. However, the series by McMillan et al. is very small; better designed and statistically powered studies are therefore needed to confirm that this novel technique is truly effective in patients undergoing ACLR.

## 12. Conclusions

Future studies are needed to assess the efficacy of BA techniques in ACLR and to determine whether a combination of various therapies could be beneficial in clinical practice. Despite the promising results obtained in animal models with all BA methods, they are still experimental. More clinical studies that meet the minimum quality standards are needed to determine whether current and emerging BA techniques in ACLR are truly beneficial to patients. Orthopedic surgeons should be very cautious when considering using BA in ACLR and should rely on proven scientific data and not get swept up by the BA fad, the clinical efficacy of which has yet to be proven.

## Figures and Tables

**Table 1:** Growth factors used in anterior cruciate ligament reconstruction.

Bone Morphogenetic Growth Proteins
Basic fibroblast growth factor
Epidermal growth factor
Granulocyte colony-stimulating factor
Hepatocyte growth factor
Transforming growth factor-β
Vascular endothelial growth factor
Platelet concentrates: +Platelet-rich plasma (PRP) +Fibrin clots +Autologous conditioned serum

**Table 2:** Stem cells used in anterior cruciate ligament reconstruction.

Adipose-Derived Stem Cells
Bone marrow-derived stem cells
Induced pluripotent stem cells
Umbilical cord-derived mesenchymal stem cells
Tendon-derived stem cells
CD34+ ACL-derived stem cells
Stem cells seeded on scaffold (in the form of sheets or applied locally to grafts)

**Table 3:** Autologous tissue used in anterior cruciate ligament reconstruction.

Autologous Tissue Over Cultured Stem Cells
Attachment of the ACL remnant to the graft: single anteromedial bundle biological augmentation technique
Periosteal grafts

**Table 4:** Drugs employed in anterior cruciate ligament reconstruction.

Matrix Metalloproteinase-Inhibitor Alpha-2-Macroglobulin: Intra-Articular Injection
Bisphosphonates (alendronate): local or systematic administration
Subcutaneous parathyroid hormone

**Table 5:** Biophysical and environmental methods used in anterior cruciate ligament reconstruction.

Hyperbaric Oxygen
Low-intensity pulsed ultrasound
Extracorporeal shockwave therapy applied to the tibial tunnel

**Table 6:** Biomaterials used in anterior cruciate ligament reconstruction.

**Biological Fixation Methods** +Magnesium-Based Interference Screws +Biodegradable Polylactide Bolts As The Bone Anchor +Poly(D,L-Lactide-Co-Glycolide) Nanofibrous Membrane At The Graft-Tunnel Interface
**Biological coatings** +Chitin +Bioglass +Gelatin and hyaluronic acid +Polystyrene sodium sulfonate: collagen matrix; hydroxyapatite-doped polycaprolactone nanofiber membrane wrapped around autograft hamstring tendons
**Biosynthetic bone substitutes** +Demineralized bone matrix +Recombinant bone xenograft (nanohydroxyapatite bone-based graft).
**Osteoconductive materials** +Calcium phosphate

**Table 7:** Gene therapies employed in anterior cruciate ligament reconstruction.

Tendon Graft Infected In Vitro With Adenovirus-BMP-2
BMP-2 gene-transfected normal rat kidney cells at the tendon-bone interface
Transfecting stem cells with growth factors such as BMP-2, platelet-derived growth factor subunit and transforming growth factor beta
Implantation of genetically modified mesenchymal stem cells with basic fibroblast growth factor and BMP-2 at the graft-tunnel interface

BMP = bone morphogenetic protein.
